# Supplementary material for: Flickering Emergences: The Question of Locality in Information-Theoretic Approaches to Emergence
Source: Entropy (Basel). 2022 Dec 28;25(1):54. doi: 10.3390/e25010054 (PMC9858457; doi:10.3390/e25010054)
Supplement: Supplementary file 1 [file entropy-25-00054-s001.zip › entropy-1949080-supplementary.pdf]

Supplementary material for "Flickering emergences"

Thomas F. Varley [1,2]

[1] Department of Psychological and Brain Sciences, Indiana University Bloomington, Bloomington, IN 47405, USA
[2] School of Informatics, Computing, & Engineering, Indiana University Bloomington, Bloomington, IN 47405, USA

Supplementary Figure

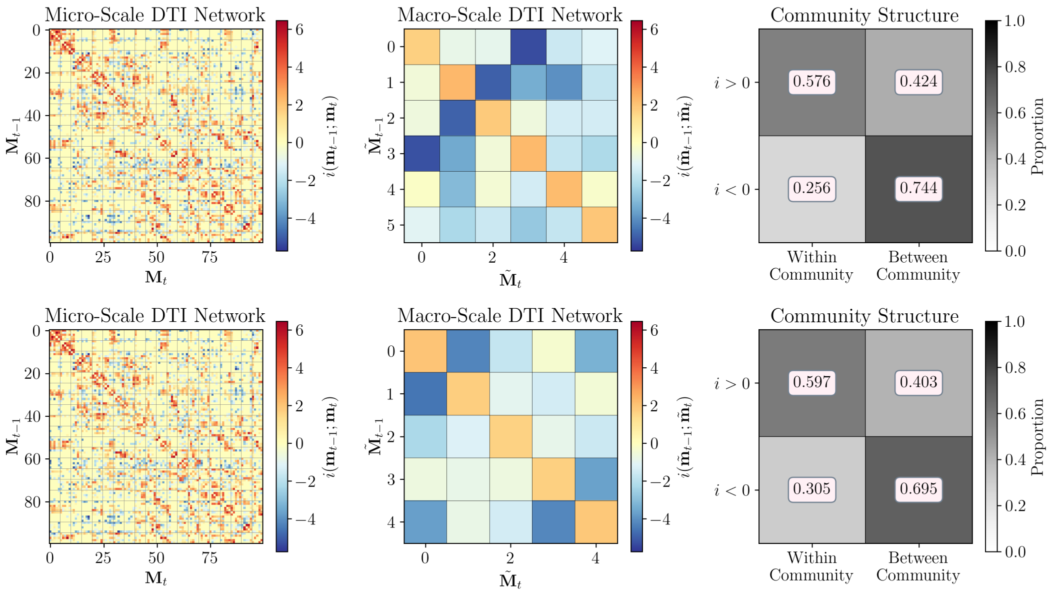

Figure S1: Replicating the network emergence results using community-detection algorithms not **based on random walkers. Top:** Spinglass [1, 2], **Bottom:** Leading eigenvector community detection.

References

[1] Jörg Reichardt and Stefan Bornholdt. Statistical mechanics of community detection. Physical Review E, 74(1):016110, July 2006. Publisher: American Physical Society.

[2] V. A. Traag and Jeroen Bruggeman. Community detection in networks with positive and negative links. Physical Review E, 80(3):036115, September 2009. Publisher: American Physical Society.
